# Covalently Conjugated Gold–Porphyrin Nanostructures

**DOI:** 10.3390/nano10091644

**Published:** 2020-08-21

**Authors:** Luca Spitaleri, Chiara M. A. Gangemi, Roberto Purrello, Giuseppe Nicotra, Giuseppe Trusso Sfrazzetto, Girolamo Casella, Maurizio Casarin, Antonino Gulino

**Affiliations:** 1 Department of Chemical Sciences, University of Catania, Viale Andrea Doria 6, 95125 Catania, Italy; luca.spitaleri@phd.unict.it (L.S.); gangemichiara@unict.it (C.M.A.G.); rpurrello@unict.it (R.P.); 2 National Interuniversity Consortium of Materials Science and Technology (I.N.S.T.M., Research Unit (UdR) of Catania, Viale Andrea Doria 6, 95125 Catania, Italy; 3 National Research Council—Institute for Microelectronics and Microsystems (CNR-IMM), Strada VIII, 5, 95121 Catania, Italy; giuseppe.nicotra@imm.cnr.it; 4 Department of Earth and Sea Sciences, University of Palermo, Via Archirafi 22, 90123 Palermo, Italy; girolamo.casella@unipa.it; 5 Department of Chemical Sciences, University of Padova, Via Francesco Marzolo 1, 35131 Padova, Italy; maurizio.casarin@unipd.it

**Keywords:** gold nanoparticle, porphyrin, surface plasmon resonance, luminescence, nanostructures

## Abstract

Gold nanoparticles show important electronic and optical properties, owing to their size, shape, and electronic structures. Indeed, gold nanoparticles containing no more than 30–40 atoms are only luminescent, while nanometer-sized gold nanoparticles only show surface plasmon resonance. Therefore, it appears that gold nanoparticles can alternatively be luminescent or plasmonic and this represents a severe restriction for their use as optical material. The aim of our study was the fabrication of nanoscale assembly of Au nanoparticles with bi-functional porphyrin molecules that work as bridges between different gold nanoparticles. This functional architecture not only exhibits a strong surface plasmon, due to the Au nanoparticles, but also a strong luminescence signal due to porphyrin molecules, thus, behaving as an artificial organized plasmonic and fluorescent network. Mutual Au nanoparticles–porphyrin interactions tune the Au network size whose dimension can easily be read out, being the position of the surface plasmon resonance strongly indicative of this size. The present system can be used for all the applications requiring plasmonic and luminescent emitters.

## 1. Introduction

Hybrid molecular–nanoparticle materials, obtained by a bottom-up approach, are well suited for the fabrication of functional nanostructures that display structural control [1] (in terms of ability to direct the formation of large assemblies in solution and in the solid state) and show well-defined properties, i.e., to obtain building blocks for molecular switches [2], systems for photocatalysis and photodynamic therapy [3], nanowires for multienzyme-cooperative antioxidative systems [4], materials able to enhance light absorption [5], and, in general, hybrid organic–inorganic materials showing desired functions [6,7,8].

Porphyrin and metalloporphyrin molecules have attracted great attention because of their interesting chemical, biological, optical, and electronic properties [9]. Moreover, surface-anchored molecular switches [10], porphyrins for charge-based information storage [11,12], and porphyrin nanostructures with a long range order on Si(100) [13] have been reported. In this context, porphyrin molecules anchored to electroactive surfaces can be used to build molecular memories that can be integrated in the electronic circuits. In fact, porphyrin-based information storage elements exhibit large charge retention times (minutes) compared to those of the semiconductor elements in dynamic random access memory (tens of milliseconds) [12].

Additionally, Au nanoparticles (Au NPs) are attracting the attention of a large community of scientists because of their tunable electronic structures that generate characteristic and modifiable chemical, optical, and electronic properties. In fact, Au NPs with large surface area-to-volume ratio display size- and shape-dependent physicochemical properties, which are of huge importance for applications in different technological fields, such as photonics, catalysis, electronics, sensors, therapeutics, etc. [14,15,16]. Actually, different particle dimensions and additional structural factors, such as aggregation and shape irregularity, may also considerably affect the overall properties, as the surface plasmon resonance that appears at different wavelengths depending on the abovementioned reasons [9,10,11,12,13,17,18,19,20,21,22,23,24,25,26,27].

Hybrid materials composed of both Au NPs and porphyrin molecules that cover their surfaces combine many of their distinctive characteristics, thus, originating unique properties fundamental for the development of optical devices that can be used for many different applications, e.g., to mimic natural photosynthesis [28,29,30,31,32,33,34].

Some different synthetic methods have been proposed to obtain these assemblies, depending on the porphyrin functional group used as a linker for the Au NPs [35,36,37,38,39,40]. For this purpose, we have synthesized a new porphyrin with two triazine moieties in two opposite positions of the porphyrin ring. This porphyrin molecule is well suited to act as a bridge for Au NPs in order to obtain an extensive and organized network of Au NPs, covalently linked by organic and emissive connections. To our knowledge, this represents one of the first few cases of an Au NPs–porphyrin organized, plasmonic, and emissive network [26,27,41,42,43,44,45].

Therefore, in the present study, we investigated the formation of a new composite assembly consisting of Au NPs covalently anchored to each other by 5,15,-Di(phenyl) 10,20-Di-benzamide, *N*-ethyl, *N*-1,3,5 Tri-aminotriazine, and 21H,23H-porphine- (DTAzDPH2P) molecules. The rationalization of the mutual interactions in this assembled nanocomposite 3D architecture will allow the addressing of applications of this novel plasmonic and emissive material.

## 2. Materials and Methods

### 2.1. Synthesis of Au NPs

All glassware used for the present synthesis was carefully washed with aqua regia (conc. HNO_3_ and HCl: 1:3 *v/v* ratio, Sigma-Aldrich, Buchs, Switzerland). A total of 0.036 g of sealed tetrachloroauric acid (HAuCl_4_·3H_2_O, MM = 393.835 g/mol, purity ≥ 99.9%, Sigma-Aldrich, Buchs, Switzerland) were dissolved in 180 mL of double distilled H_2_O (concentration of this stock solution 5.08 × 10^−4^ M, mols of Au^3+^ = 9.14 × 10^−5^). Then, we added 18 mL of this Au^3+^ stock solution to a three-necked flask, equipped with a bubble cooler, and brought it to boil on a heating plate, while stirring. Meanwhile, 0.014 g of sodium citrate dihydrate (Na_3_C_6_H_5_O_7_·2H_2_O, MM = 294.10 g/mol, Sigma-Aldrich, Buchs, Switzerland) were dissolved in 2 mL of fresh, double distilled H_2_O (2.38 × 10^−2^ M, mols of Cit^3−^ = 4.76 × 10^−5^) and added to the boiling tetrachloroauric acid solution while stirring, thus, obtaining a Cit^3−^/Au^3+^ ratio = 5.21 [46]. This solution was refluxed 20 min and its color turned to ruby red, typical of Au NPs in water.

### 2.2. Synthesis of Di-Triazine-Porphyrin

**Scheme 1:**
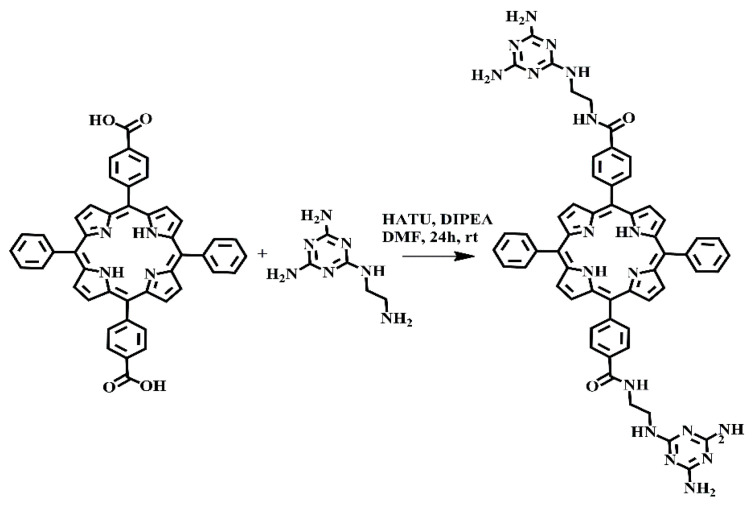
Reaction pathway for the Di-Triazine-Porphyrin.

The functionalization of the porphyrin was performed by a synthetic procedure modified as follows [47,48,49]: 0.14 mmol of 5,15-di-(p-carboxyphenyl)-10,20-diphenyl porphyrin [50] (Sigma-Aldrich, Buchs, Switzerland), 0.336 mmol of 1-[Bis(dimethylamino)methylene]-1H-1,2,3-triazolo [4,5-b]pyridinium 3-oxid hexafluorophosphate, N-[(Dimethylamino)-1H-1,2,3-triazolo-[4,5-b]pyridin-1-ylmethylene]-N-methylmethanaminium hexafluorophosphate N-oxide (HATU, a coupling reagent for the synthesis of amides, Merck, Milan, Italy) were dissolved in 10 mL of dry DMF (Sigma-Aldrich, Buchs, Switzerland) under N_2_ atmosphere (Scheme 1). Then, 0.427 mmol of N^2^-(2-aminoethyl)-1,3,5-Triazine-2,4,6-triamine (Sigma-Aldrich, Buchs, Switzerland) [51] and 58 µL of N,N-Diisopropylethylamine (DIPEA, an organic base, Sigma-Aldrich, Buchs, Switzerland) were added to the mixture. The reaction mixture was stirred at room temperature for 24 h under nitrogen and then, poured in cooled n-hexane (Sigma-Aldrich, Buchs, Switzerland), thus, obtaining a brown precipitate which was filtered and washed with n-hexane. The Di-Triazine-Porphyrin was purified by flash chromatography (Al_2_O_3_, CHCl_3_:CH_3_OH 95:5, Sigma-Aldrich, Buchs, Switzerland) and further crystallized by ethanol (yield 51%). ^1^H NMR (Varian UNITY Inova 500 MHz, CA, USA, DMSO-*d_6_*)—2.91 (s, 2H, NH-Pyr), 3.31 (m, 4H, CH_2_), 3.61 (m, 4H, CH_2_), 7.42 (br. s. 8H, NH_2_-triazine), 7.85 (m, 6H, meso-ArH), 7.89 (br. 2H, NH-triazine), 8.22 (d, J = 6 Hz, 4H, meso-ArH), 8.31 (m, 8H, meso-ArH-triazine), 8.84 (m, 8H, β-Pyr), and 8.94 (s. br. 2H, NH amide) ppm (Figures S1–S4). ESI-MS: *m*/*z* 1005.8 [M + H]^+^; *m*/*z* 503.5 [M + 2H]^2+^ (Figure S5). Anal. Calcd. for C_56_H_48_N_18_O_2_: C, 66.92; H, 4.81; N, 25.08. Found: C, 66.88; H, 4.77; N, 25.01.

### 2.3. Syntheses of the Au NPs–Porphyrin Nanostructures

**Scheme 2:**
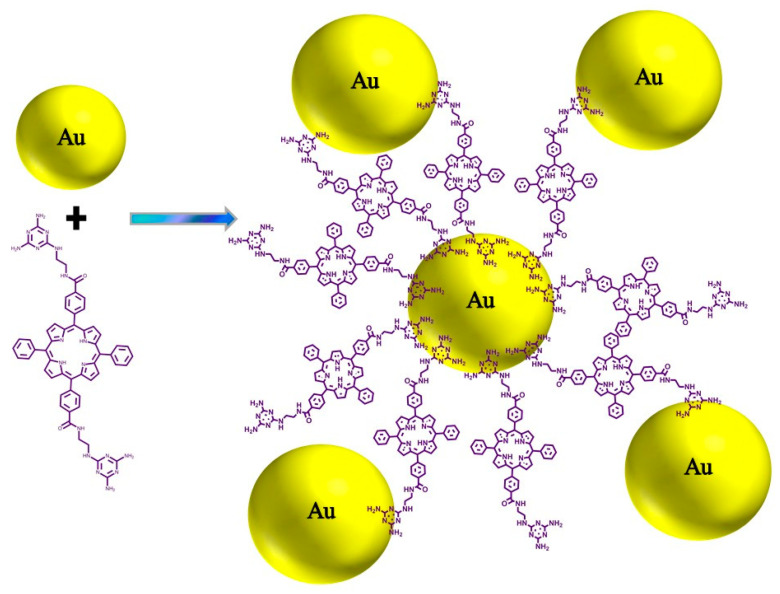
Reaction pathway for the Au NPs–porphyrin nanostructures.

In order to synthesize the Au NPs–porphyrin nanostructures, we added aliquots of a 2.07 × 10^−5^ M porphyrin CH_3_OH solution (final added volume 900 µL, in 400 min, corresponding to 1.86 × 10^−8^ mol) to 100 µL of an Au NPs aqueous 1.97 × 10^−7^ M solution, diluted with 2 mL of CH_3_OH (final total volume 3 mL) [52]. The synthetic reaction pathway is represented in Scheme 2.

In this proposed scheme, the triazine moieties, which are at 180° with respect to the porphyrin core, covalently bind different Au nanoparticles. The possibility of back-folding of the porphyrin substituents, with both legs attached to the same Au NPs, was investigated by accurate quantum mechanical calculations (vide infra) and resulted in being unlikely. Moreover, quantum mechanical calculations used to optimize the grafting geometry of similar polyfunctional porphyrin molecules on surfaces always indicated geometries tilted with respect to the normal to the surface plane but reminiscent of perpendicular arrangements [13].

### 2.4. Transmission Electron Microscopy Measurements

Transmission Electron Microscopy (TEM) measurements were performed using an Atomic Resolution Analytical Microscope (JEOL ARM200F Cs-corrected, JEOL USA, Peabody, Massachusetts, MA, USA). The samples were placed on a Cu/C TEM Grid. S/TEM and Energy Dispersive X-ray (EDX) chemical analyses were performed using a 60 K eV electron beam.

### 2.5. X-ray Photoelectron Data

X-ray photoelectron spectra (XPS) were measured with a PHI 5600 Multi Technique System (Physical Electronics GmbH, Feldkirchen, Germany, base pressure of the main chamber 3 × 10^−8^ Pa) [53,54,55]. Samples, placed on a molybdenum specimen holder, were excited with Al-Kα X-ray radiation using a pass energy of 5.85 eV. The instrumental energy resolution was ≤0.5 eV. Structures due to the Al-Kα X-ray satellites were subtracted prior to data processing. XPS peak intensities were obtained after Shirley background removal. Spectra calibration was achieved by fixing the Ag 3d_5/2_ peak of a clean sample at 368.3 eV; this method turned the C 1s main peak at 285.0 eV [53,54]. The atomic concentration analysis was performed by taking into account the relevant atomic sensitivity factors. The fitting of the XP spectra in the C1s and N 1s binding energy regions was carried out by fitting the spectral profiles with symmetrical Gaussian envelopes, after subtraction of the background. This process involves data refinement, based on the method of the least squares fitting, carried out until there was the highest possible correlation between the experimental spectrum and the theoretical profile. The residual or agreement factor *R*, defined by *R* = [Σ (F_obs_ − F_calc_)^2^/Σ (F_obs_)^2^]^1/2^, after minimization of the function Σ (F_obs_ − F_calc_)^2^, converged to the value of 0.03. The fitting was performed using the XPSPEAK4.1 software. XPS measurements were performed on the centrifuged Au NPs–porphyrin material.

### 2.6. UV–vis Spectra

UV–vis measurements of 100 µL of an Au NPs aqueous 1.97 × 10^−7^ M solution diluted with 2 mL of CH_3_OH upon the addition up to 900 µL of a 2.07 × 10^−5^ M porphyrin CH_3_OH solution were carried out using a UV–vis V-650 Jasco spectrometer (UV–Visible Spectrometers, Easton, MD, USA). The spectra were recorded with a 0.2 nm resolution at room temperature in quartz cells with a path length of 1 cm (3.5 mL capacity). 

### 2.7. PL Measurements

Luminescence measurements of 100 µL of an Au NPs aqueous 1.97 × 10^−7^ M solution diluted with 2 mL of CH_3_OH upon the addition up to 900 µL of a 2.07 × 10^−5^ M porphyrin CH_3_OH solution were carried out using a Varian Cary Eclipse fluorescence spectrophotometer (Varian Optical Spectroscopy Instruments, Mulgrave, Victoria, Australia) with a λ_exc_ of 410 nm at 1 nm resolution and at room temperature in quartz cells with a path length of 1 cm (3.5 mL capacity). The emission was recorded at 90° with respect to the exciting line beam using 5:5 slit widths.

The “footprint” of the porphyrin molecule was calculated by a Molecular Mechanics optimization, with the HypChem^TM^ (v8.0.7. Gainesville, FL, USA) code, using the so-called MM+ as the force field. This software optimizes the molecular geometry (length and angle bonds) to obtain a minimum of the total energy. In addition, non-covalent interactions such as hydrogen bonds, van der Waals interactions, steric hindrance, and electrostatic interactions were included. In our case, we used a conjugated gradient (Polak–Ribière) to obtain a minimum of energy, setting the end of the optimization when the gradient energy between the optimized structure was lower than 0.01 kcal/mol.

### 2.8. Quantum Mechanical Calculation

**Figure 1:**
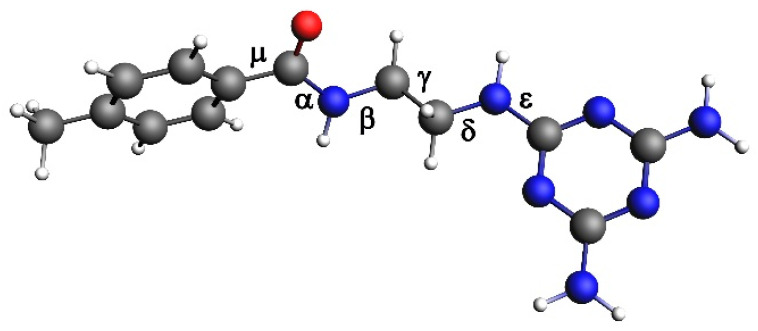
Labeling of the bonds for which the PES scan has been performed. μ represents the bond with the meso substituent.

All DFT calculations have been performed with the G09 package [56]. Geometry optimizations have been carried out at the B3LYP/6-31G(d, p) level of theory. Similarly, the Potential Energy Surface (PES) scans of the α–ε bonds set (see Figure 1) have been performed by considering 10 scans of 36° each [56].

## 3. Results

In this study, we preliminary reduced Au^3+^ with sodium citrate to obtain Au NPs and synthesized a new porphyrin to fabricate assemblies comprising of Au NPs covalently anchored to each other by porphyrin molecules (Scheme 2) [46,47,48,49,50,51,52].

### 3.1. Results of Quantum Mechanical Calculations

The self-assembly geometry of this porphyrin on the Au NPs (Scheme 2) was studied by investigation of the conformational properties of the porphyrin derivative at the DFT level. In particular, in order to check the potential conformations of the triazine-based substituents, we performed a Potential Energy Surface scan of the rotational barriers involved along the chain. To avoid long computational times, we have considered the model reported in Figure 1, where also the labeling of the investigated bonds is given. Indeed, the presence of the meso aromatic spacer allows the safe consideration of a negligible contribution of the porphyrin ring on the rotational barriers.

**Figure 2:**
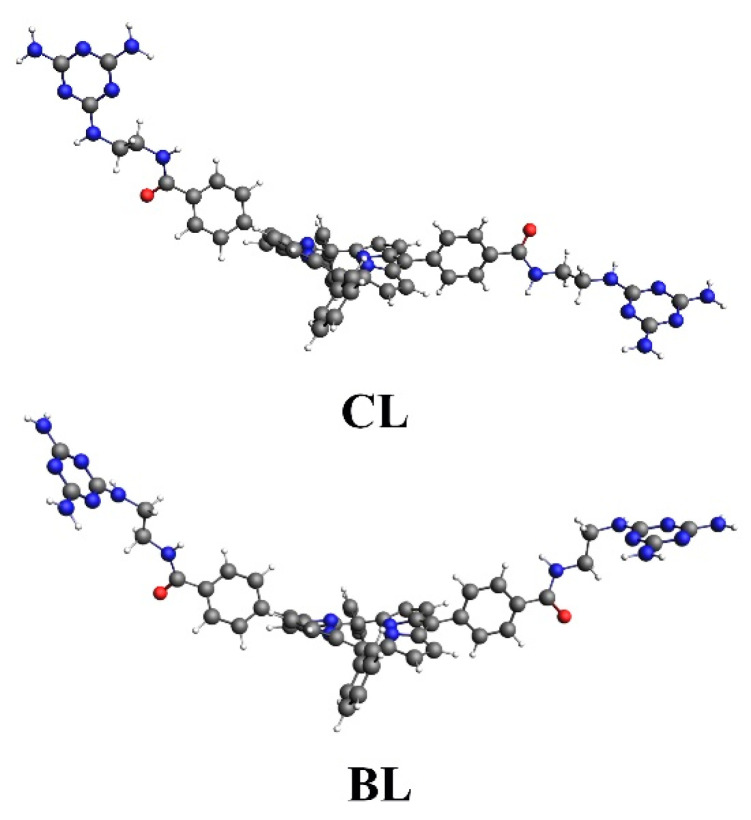
Starting geometries of the chair-like (CL) and as boat-like (BL) conformations.

As expected, rotations on the α and ε bonds are hampered, at standard conditions, by energy barriers around 17–20 kcal/mol. Nonetheless, rotations around the μ, β, γ, and δ bonds require about 4–6 kcal/mol, which indicates the possibility of a virtually free rotation around these bonds. This flexibility allows the inference of the easy occurrence of different conformations of the porphyrin derivative. In this context, two main typologies of conformations have been considered as opposite conformational paradigms. They are shown in Figure 2 and are labeled as chair-like (CL) and as boat-like (BL) conformations, respectively.

**Figure 3:**
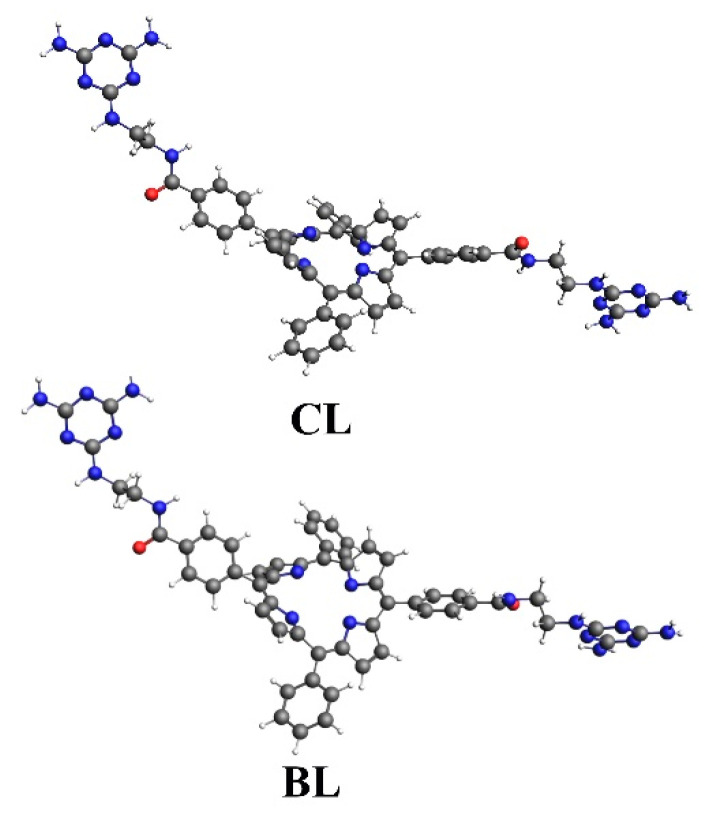
Optimized geometries of the chair-like (CL) and boat-like (BL) conformations.

CL conformation is recognized as suitable to arrange the framework in Scheme 2, hence, able to coordinate different Au NPs. In the BL conformation, by the back-folding of the porphyrin substituents, the two substituents can virtually act as a tweezer by bonding to just one Au NP. On these grounds, we optimized the corresponding structures by considering the starting geometries reported in Figure 2. The optimized geometries are reported in Figure 3 and indicate that the CL conformation tends to be the more preferred. Indeed, the optimized BL conformation, Figure 3, displayed a configuration similar to that of the CL one (see also their optimized cartesian coordinates as Supplementary Material), but, the optimized CL conformer resulted 1.6 kcal/mol more stable than the optimized BL one. A Boltzmann analysis of the population ratio indicated that CL represents 95% of the total population among these two conformers. To summarize, though the porphyrin substituents have some rotational degrees of freedom (bonds μ, β, γ, δ, in Figure 1), the CL conformation is more likely. This result corroborates our reaction path in Scheme 2.

### 3.2. XPS Results

The study of the electronic structure of the Au NPs–porphyrin nanostructures is fundamental to investigate the Au–porphyrin electron interactions, which are the basis of the coupling of the plasmon Au resonance with the porphyrin emission, and XPS represents the most suited tool to accomplish this task [34,52,53,54,55].

**Figure 4:**
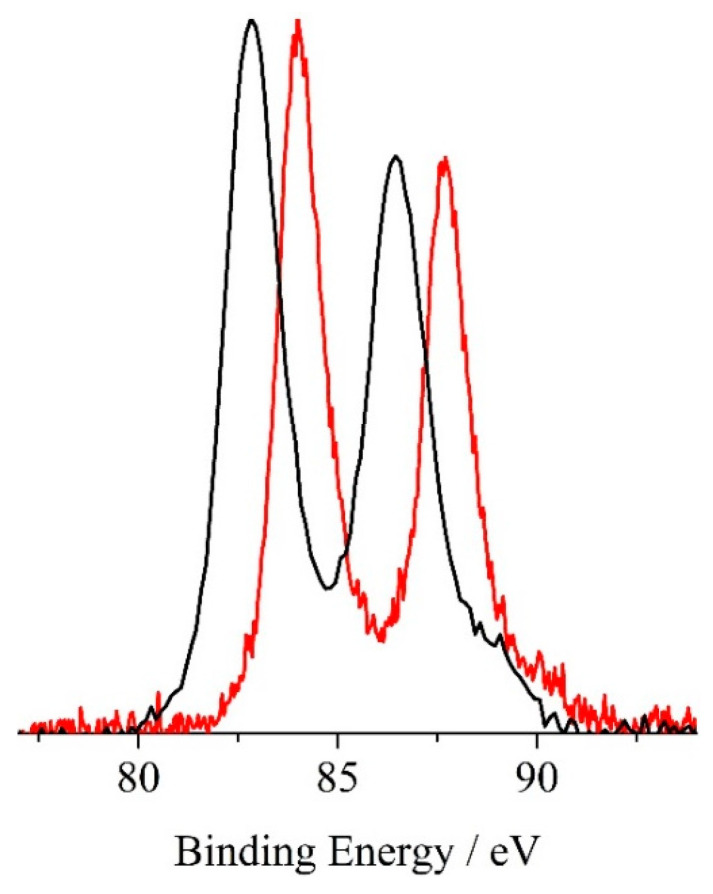
Al-Kα excited XPS of the pure Au NPs solution (red line) and Au NPs–porphyrin system (black line) in the Au 4f binding energy region.

Figure 4 shows the XP spectra of pure Au NPs and Au NPs–porphyrin nanostructures, in the Au 4f binding energy region. The 4f_7/2,5/2_ levels for the Au NPs before any porphyrin addition were observed at 84.0 and 87.7 eV, respectively [5,6]. These states lie at 82.8 and 86.5 eV (3.7 eV spin-orbit coupling), respectively, for the Au NPs–porphyrin nanostructures, and indicate the presence of Au^0^ states. Therefore, the considerably decreased values are in tune with the strong electron-donating capability of this di-triazine porphyrin to the positively charged Au NPs surfaces.

**Figure 5:**
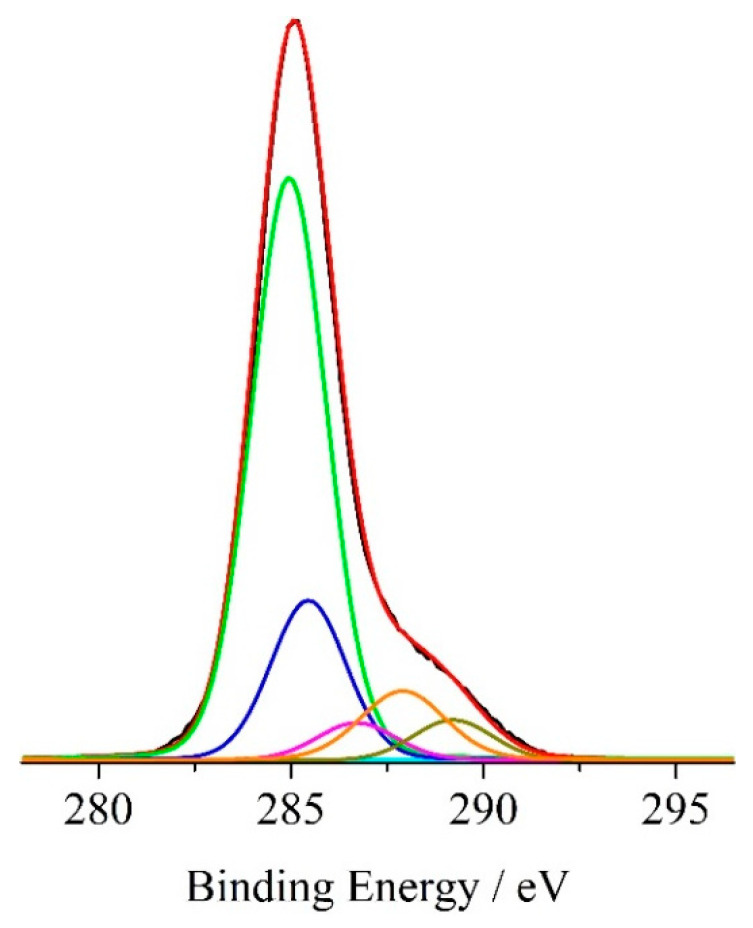
Al-Kα excited XPS of the Au NPs–porphyrin system in the C 1s binding energy region. The green, blue, magenta, orange, and dark yellow lines refer to the 285.0, 285.5, 286.7, 287.9, and 288.9 eV components; the cyan line refers to the background and the red line superimposed on the experimental black profile refers to the sum of all Gaussian components.

Figure 5 shows the XPS of the Au NPs–porphyrin system in the C 1s binding energy region. An accurate fitting of this spectrum required five Gaussian components centered at 285.0, 285.5, 286.7, 287.9, and 288.9 eV. The first component (285.0 eV) is due to both aliphatic and aromatic backbones [53,54,55]. The peaks at 285.5 and 286.7 eV are assigned to the C–N and C=N groups, respectively [55,57]. The peak at 287.9 eV is assigned to the HN-C=N(-NH) and N-C=N(-NH_2_) triazine groups [57,58]. The peak at 288.9 eV is assigned to the carbon of the amide group (Ar-CO-NH) [57].

**Figure 6:**
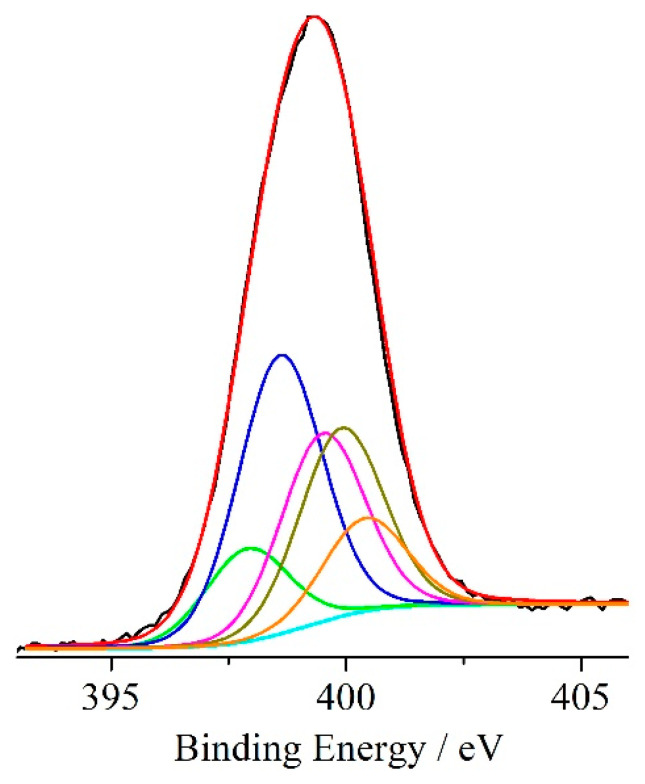
Al-Kα excited XPS of the Au NPs–porphyrin system in the N 1s binding energy region. The orange, dark yellow, magenta, blue, and green lines refer to the 400.4, 399.9, 399.5, 398.6, and 397.9 eV components; the cyan line refers to the background and the red line superimposed on the experimental black profile refers to the sum of all Gaussian components.

Figure 6 shows the XPS of the Au NPs–porphyrin system in the N 1s binding energy region. A careful fitting of the experimental profile required five Gaussian components centered at 397.9, 398.6, 399.5, 399.9, and 400.4 eV. The component at 397.9 eV is assigned to the ionization of the two non-protonated imine nitrogens of the porphyrin core, that at 398.6 eV to the six triazine ring nitrogen atoms, that at 399.5 eV is assigned to the four –NH_2_ triazine substituents, that at 399.9 eV is assigned to the two protonated pyrrole nitrogens of the porphyrin core and to the two –NH- groups bound to the triazine moiety and, finally, that at 400.4 eV is consistent with the two O=C(Ar)-NH- amide functionalities [5,55,57,58,59,60,61,62].

### 3.3. TEM Measurements

**Figure 7:**
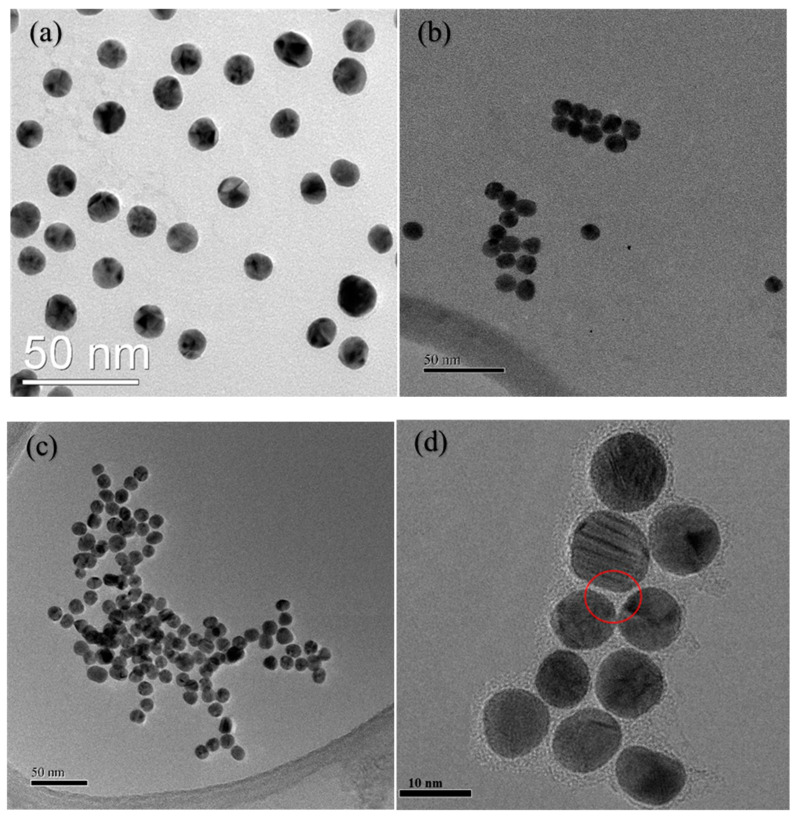
TEM images of conjugated gold–porphyrin nanostructures: (**a**) Au nanoparticles obtained from the reduction of the tetrachloroauric acid with sodium citrate; (**b**) Au NPs obtained from 100 µL of an Au NPs aqueous 1.97 × 10^−7^ M solution diluted with 2 mL of CH_3_OH upon the addition of 240 µL of a 2.07 × 10^−5^ M porphyrin CH_3_OH solution; (**c**) Au NPs obtained from 100 µL of an Au NPs aqueous 1.97 × 10^−7^ M solution diluted with 2 mL of CH_3_OH upon the addition of 450 µL of a 2.07 × 10^−5^ M porphyrin CH_3_OH solution; (**d**) higher magnification of the Au NPs obtained from 100 µL of an Au NPs aqueous 1.97 × 10^−7^ M solution diluted with 2 mL of CH_3_OH upon the addition of 450 µL of a 2.07 × 10^−5^ M porphyrin CH_3_OH solution; the red circle represents the representative area investigated with EDX to obtain Figure S6.

The TEM microscopy of the conjugated Au NPs is reported in Figure 7. While single and highly dispersed Au nanoparticles, having a mean radius of about 5 nm, have been obtained from the reduction of the tetrachloroauric acid with sodium citrate (Figure 7a), large organized (even though not apparently ordered) nanoscale assemblies of Au nanoparticles are evident in the presence of 5,15,-Di(phenyl) 10, 20-Di-benzamide, *N*-ethyl, *N*-1,3,5 Tri-aminotriazine, and 21H,23H-porphine molecules (Figure 7b,c). The role of this porphyrin in the formation of the new composite assembly, consisting of Au NPs covalently anchored to each other, is evident in Figure 7d, where the ~3 nm texture surrounding the Au NPs represents the “glue” for the covalent assembly of gold nanoparticles. We analyzed the chemical composition of these assemblies by EDX (a representative result is reported in the Supplementary Figure S6) and the results are indicative of Au nanoparticles surrounded by a thin layer of a nitrogen-containing compound, consistent with a ~3 nm, layer of porphyrin molecules. Therefore, the bi-functional porphyrin molecules work as covalent bridges between different gold nanoparticles.

### 3.4. Optical Measurements

The main purpose of our study was the nanoscale self-assembly of Au NPs by means of porphyrin molecules having two functional groups in opposite positions, useful to connect these Au NPs each other. It is well known that Au NPs can be either luminescent or plasmonic and this hampers many possible applications as optical material. Therefore, the goal we would achieve with this final functional architecture is not only to maintain the strong surface plasmon resonance, typical of Au nanoparticles, but also the conservation of a strong luminescence signal, coming from porphyrin molecules. Additionally, this property is strongly related to the absence of porphyrin aggregates which causes luminescence quenching. Obviously, we can observe and follow variations of the Au NPs surface plasmon resonance with absorbance spectra and detect the porphyrin fluorescence with emission measurements.

**Figure 8:**
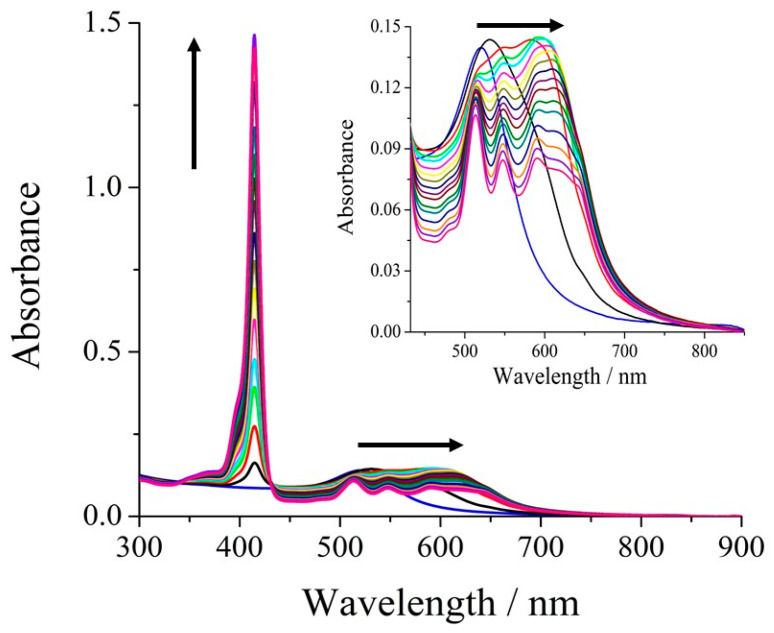
UV–vis absorbance spectra of 100 µL of an Au NPs aqueous 1.97 × 10^−7^ M solution diluted with 2 mL of CH_3_OH (blue line), and upon the addition of 40 (black line), 80 (red line), 120 (green line), 160 (cyan line), 200 (magenta line), 240 (yellow line), 280 (dark yellow line), 320 (navy line), 360 (purple line), 400 (wine line), 450 (olive line), 500 (dark cyan line), 600 (royal line), 700 (orange line), 800 (violet line), and 900 (pink line) µL aliquots of a 2.07 × 10^−5^ M porphyrin CH_3_OH solution (final total volume 3 mL). All the spectra were corrected for the volume variation. Inset: expanded scale of the Au NPs plasmon–porphyrin Q-bands region.

Figure S7, (in Supplementary), shows the UV–vis spectrum of the as synthesized aqueous Au NPs solution and Figure S8 shows UV–vis absorbance spectra of porphyrin CH_3_OH solutions at different concentrations. The Au surface plasmon resonance (SPR) peak lies at 520.6 nm (Abs = 1.5). By using literature data for the related extinction coefficient (ε = 7.6 × 10^6^ M^−1^ cm^−1^ [52]), we obtained a 1.97 × 10^−7^ M concentration value for this Au NPs solution. Figure 8 shows the absorbance spectra of 100 µL of this Au NPs aqueous solution, diluted with 2 mL of CH_3_OH (V_tot_ = 2.1 mL), and those upon the successive addition of aliquots of a 2.07 × 10^−5^ M porphyrin CH_3_OH solution, up to a final added porphyrin volume of 900 µL, corresponding to 1.86 × 10^−8^ mol, (final total volume 3 mL). The Au SPR peak, before any porphyrin addition, almost does not move (520.4 nm, Abs = 0.14) with respect to the position observed for the aqueous solution, but the calculated extinction coefficient now is ε = 1.5 × 10^7^ M^−1^ cm^−1^ for this Au NPs CH_3_OH solution, almost double, once the dilution from 100 µL up to 2.1 mL is taken into account.

As the titration goes on, it is evident the progressive and monotonic increase in the Soret at 414.8 nm and Q-bands at 513.6, 547.8, 590.6, and 642.0 nm. The Soret band of a CH_3_OH 2.016 × 10^−6^ M pristine porphyrin solution (absorbance = 0.60, ε = 289,300 M^−1^ cm^−1^) appears at 414.4 nm and the related Q-bands are at 512.8, 546.8, 589.2, and 644.0 nm, thus, indicating about 1 nm red shift upon the interaction with the Au NPs. Therefore, the light-induced surface plasmon resonance, coherent collective oscillation of the valence electrons, and porphyrin absorptions result in an intense band over a wide wavelength range and this system can mediate excitation energy transport, e.g., to mimic a natural “light-harvesting” function.

The concentration of the porphyrin in the final solution containing the self-assembled Au NPs–porphyrin nanostructures is 6.22 × 10^−6^, about 950 times larger than that of the Au NPs (100 μL 1.97 × 10^−7^ M diluted to 3 mL = 6.57 × 10^−9^ M), thus, indicating the possibility of a total Au surface coverage with porphyrin molecules. In fact, each Au nanoparticle with a ~5 nm radius has a surface area of 314 nm^2^ (31400 Å^2^), while the footprint of the porphyrin molecule, assumed perpendicular to the Au NP surface (95% of the total porphyrin population) and calculated with a MM+ method, is about 65 Å^2^, thus, confirming the possibility to accommodate about 480 porphyrin molecules per Au nanoparticle. Therefore, this 480/1 porphyrin/Au NP ratio was obtained upon the addition of 450 µL of the porphyrin solution to the starting Au NPs aqueous 1.97 × 10^−7^ M solution diluted with 2 mL of CH_3_OH. It is important to note that in these exact conditions, we observed the maximum emission intensity of the Au NPs–porphyrin system (vide infra). The literature data show that the binding of Au NPs with luminescent dyes could lead to strong coupling of the plasmonic mode with molecular modes, but, in the present case, no peaking splitting was observed because of the mismatch resonance peak between AuNP and the porphyrin molecule [63,64,65].

**Figure 9:**
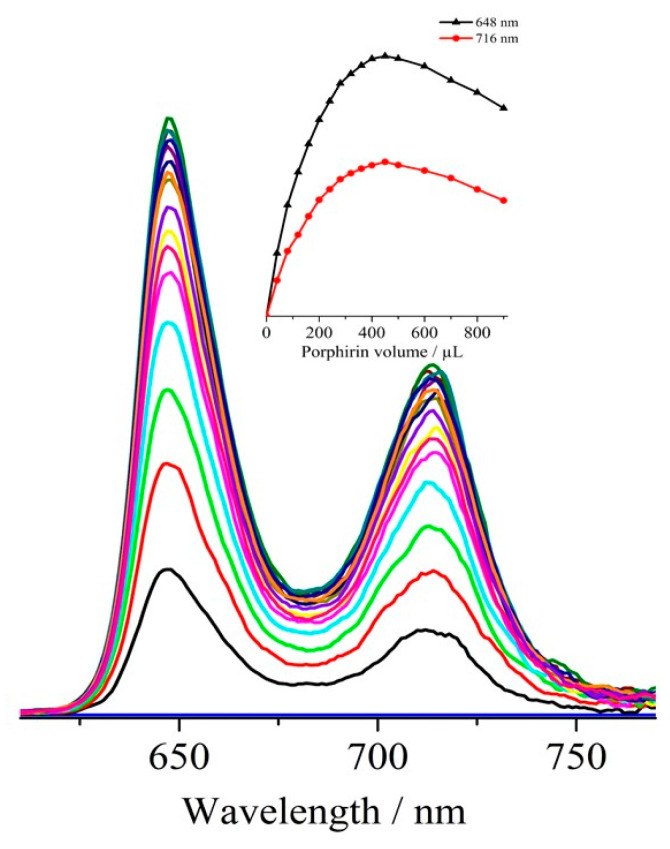
Fluorescence spectra (λ_ex_ = 410 nm) of 100 µL of an Au NPs aqueous 1.97 × 10^−7^ M solution diluted with 2 mL of CH_3_OH (blue line superimposed to the *x*-axis), 40 (black line), 80 (red line), 120 (green line), 160 (cyan line), 200 (magenta line), 240 (yellow line), 280 (dark yellow line), 320 (navy line), 360 (purple line), 400 (wine line), 450 (olive line), 500 (dark cyan line), 600 (royal line), 700 (orange line), 800 (violet line), and 900 (pink line) µL aliquots of a 2.07 × 10^−5^ M porphyrin CH_3_OH solution (final total volume 3 mL). All the spectra were corrected for the volume variation. Inset: intensity behavior of the emission maxima upon the porphyrin additions.

Figure 9 shows the emission spectra of the above solution (100 µL of an Au NPs aqueous 1.97 × 10^−7^ M solution diluted with 2 mL of CH_3_OH, and those upon the successive addition of aliquots of a 2.07 × 10^−5^ M porphyrin CH_3_OH solution, up to a final added porphyrin volume of 900 µL). Figure S9 shows the luminescence spectra of the porphyrin CH_3_OH solution at different concentrations and Figure S10 shows a comparison between the PL intensities of the porphyrin CH_3_OH solutions and those obtained during the Au NPs titration at different concentrations. The emission intensities in Figure 9 of both 648 and 715 nm bands reach maximum values upon the addition of 450 µL of the porphyrin solution (9.3 × 10^−9^ mol; 3.652 × 10^−6^ M) and in these conditions, the porphyrin concentration corresponds to that for the total Au surface coverage. It is also important to stress that at each porphyrin concentration obtained in our experiments, the porphyrin emission intensity was always lower than that observed for pure porphyrin solutions with the same concentrations. In fact, after the addition of 240 and 450 µL of the porphyrin solution, we noted 16% and 34% emission decrease, respectively.

**Figure 10:**
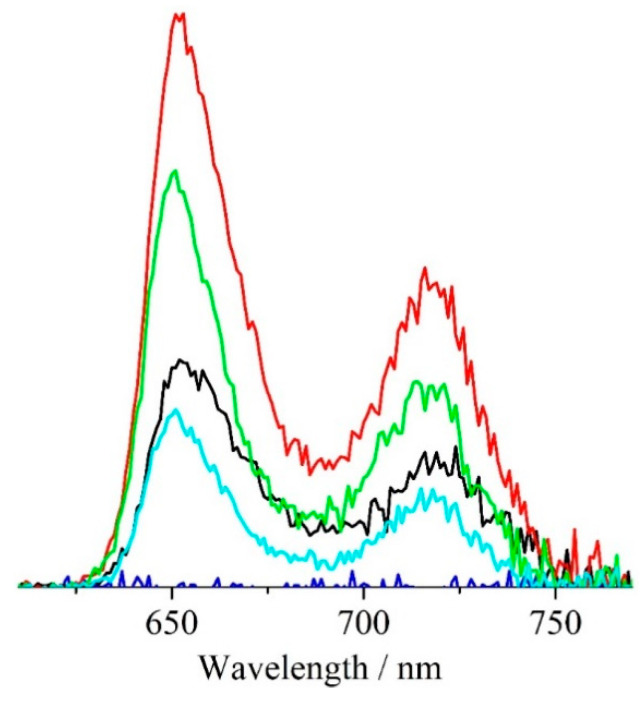
Fluorescence spectra (λ_ex_ = 410 nm) of the Au NPs–porphyrin systems centrifuged and deposited on quartz substrates. Starting Au NPs (blue line), centrifuged solutions upon the addition of 240 (black line), 450 (red line), 700 (green line), and 900 (cyan line) µL aliquots of a 2.07 × 10^−5^ M porphyrin CH_3_OH solution (final total volume of the solution 3 mL).

An identical behavior was observed for the centrifuged Au NPs systems deposited on quartz substrates (Figure 10), thus, stressing that the luminescence is maintained at the solid state and that the maximum luminescence is observed upon the addition of 450 µL of a 2.07 × 10^−5^ M porphyrin CH_3_OH solution to 100 µL of an Au NPs aqueous 3.66 × 10^−7^ M solution diluted with 2 mL of CH_3_OH (final total volume of the solution 3 mL).

## 4. Discussion

First of all, we want to discuss the geometry assumed by the porphyrin in the self-assembly with the Au NPs. In this context, we performed accurate quantum mechanical calculations with a Gaussian Code to check the potential conformations of the triazine based substituent and performed a Potential Energy Surface scan of the rotational barriers (Figure 1). Specifically, we optimized the corresponding structures by considering the starting geometries of two main conformations labeled as chair-like (CL) and boat-like (BL) (Figure 2). The optimized geometries (Figure 3) indicated that the CL conformation tends to be the more preferred and the related Boltzmann analysis of the population ratio indicated that CL represents 95% of the total population among these two conformers. Therefore, though the porphyrin substituents have some rotational degrees of freedom, the CL conformation is more likely, and this result corroborates our assumptions shown in Scheme 2. As a consequence, the possibility of the back-folding of the porphyrin substituents so that the two substituents can virtually act as a tweezer by bonding to just one Au NP may be related just to 5% of the porphyrin molecules.

The photoluminescence of emitting dyes chemically bound to Au NPs has already been explored [66,67,68,69,70,71]. Results are always indicative of pronounced fluorescence quenching of the given dyes. In some studies, it emerged that quenching was caused not only by an increased nonradiative rate, but equally important, by a drastic decrease in the dye’s radiative rate [66]. Additionally, reduced fluorescence, for particular Au NPs–dye distances, almost exclusively governed by a phase-induced suppression of the radiative rate, has been shown [67]. This behavior has been confirmed and the strongly distance-dependent fluorescence quenching in Au NPs covered with some polyelectrolytes has been ascribed to the fact that gold nanoparticles decrease the transition probability for radiative transitions [68]. Furthermore, it has been reported that the quenching behavior may be consistent with 1/d^4^ separation distance from dye to the surface of the nanoparticle and that energy transfer to the metal surface is the dominant quenching mechanism [69]. Therefore, to maintain the dye’s emission intensity, Au NPs and the emissive dye should be distant [70]. Consequently, in our system, upon the addition of 450 µL of the porphyrin solution, the porphyrin molecules extensively reticulate with the gold nanoparticles to produce an organized Au NPs–porphyrin network and this partially quenches the porphyrin emission because the Au NPs and porphyrin molecules are close each other. In addition, this experimental observation confirms that the porphyrin molecules bond different Au NPs, since, if a relevant number of porphyrin molecules would back-fold and bond the same Au NPs, the emission quenching would be severe because of the reduced Au NPs–porphyrin distance. This quenching is rather a plasmonic quenching effect as already observed in many cases where the Au NPs are mixed with fluorescent molecules. In fact, (vide infra), the porphyrin molecules alone do not show any concentration-dependent aggregation nor quenching of absorption and luminescence at the concentrations used in the present experiments and, in contrast, in this Au NPs–porphyrin system, we noted an increase in the porphyrin ε value (vide infra). As a result, a delicate balance of Au NPs and porphyrin concentration will allow the synthesis of an organized Au network that remains plasmonic and emissive, and we observed the maximum emission intensity upon the addition of 450 µL of the porphyrin solution, exactly that needed for the total Au surface coverage. After the addition of 450 µL of the porphyrin solution, all Au NPs are covered with porphyrins and the decrease in luminescence intensity, observed upon further porphyrin addition, is now due to some possible interactions between the porphyrin molecules in solution that can interact with those linked to Au NPs and be partially responsible for the observed optical behavior.

We are aware that time-resolved fluorescence experiments could allow further insight into this behavior, but they are out of the scope of the present study.

**Figure 11:**
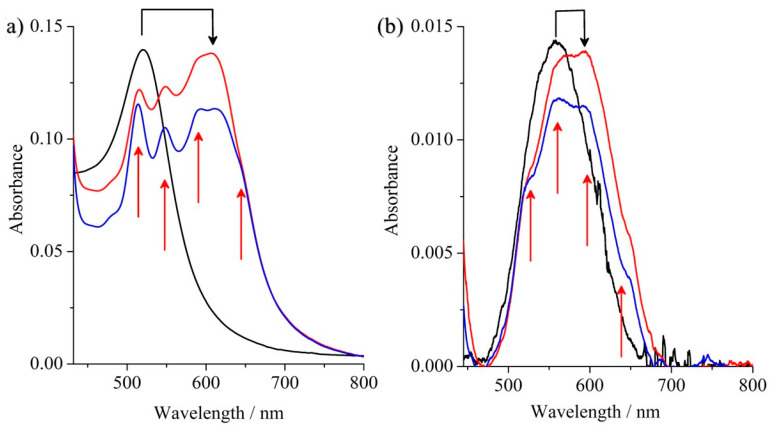
UV–vis absorbance spectra of (**a**) 100 µL of an Au NPs aqueous 1.97 × 10^−7^ M solution diluted with 2 mL of CH_3_OH (black line), upon the addition of 240 µL of a 2.07 × 10^−5^ M porphyrin CH_3_OH solution (red line, corrected for the volume variation), and upon the addition of 450 µL of a 2.07 × 10^−5^ M porphyrin CH_3_OH solution (blue line, corrected for the volume variation), and of (**b**) the centrifuged same Au NPs–porphyrin solution deposited on quartz substrates, in the 430–850 nm wavelength range. The red arrows indicate the four Q-bands at 513.6, 547.8, 590.6, and 642.0 nm and the black arrow indicates the Au NPs surface plasmon resonance that, in solution, moved from 520.4 to 612 nm and on quartz substrates moved from 559 to 595 nm.

Figure 11a shows three selected spectra of Figure 8, in particular, the absorbance spectrum of 100 µL of an Au NPs aqueous 1.97 × 10^−7^ M solution diluted with 2 mL of CH_3_OH (black line), that after the addition of 240 µL of a 2.07 × 10^−5^ M porphyrin CH_3_OH solution (red line), and that after the addition of 450 µL of this porphyrin CH_3_OH solution (blue line). The comparison of these three spectra reveals that the first two porphyrin Q-bands are evident and slightly affected by the rather broad Au NPs surface plasmon resonance that, in contrast, moved to 612 nm (already upon the addition of 240 µL of porphyrin), thus, overlapping with the two higher wavelength Q-bands. In principle, this red-shift of the Au plasmon may be due either to aggregation or a strong coupling of the two systems, but the Au NPs–porphyrin bonding distance is not close enough for a strong coupling, being the length (distance between the two opposite triazine moieties) of the porphyrin molecule ~30 Å [63,64,65]. In this condition (porphyrin conc. 1.95 × 10^−6^ M), the porphyrin shows an ε value of 353,800 M^−1^ cm^−1^ with an increase of 22%, with respect to the starting 289,300 M^−1^ cm^−1^ value (for the 2.016 × 10^−6^ M solution). Since it is well known that porphyrin aggregation causes a decrease in the molar extinction coefficient, this experimental observation strongly suggests that, after the addition of 240 µL of a 2.07 × 10^−5^ M porphyrin CH_3_OH solution to the Au NPs solution, there are no porphyrin aggregates in solution and all these molecules are involved in the formation of the Au NPs–porphyrin nanostructures. After the addition of 450 µL of a 2.07 × 10^−5^ M porphyrin CH_3_OH solution to that of the Au NPs, we just noted an overall absorbance intensity decrease (blue line). A similar trend was observed for the centrifuged same Au NPs–porphyrin solutions deposited on quartz substrates (Figure 11b), being the starting Au NPs plasmon resonance at 559 nm and that after the addition of 450 µL of the porphyrin CH_3_OH solution at 595 nm. It is important to point out that, in these conditions, we have obtained Au NPs–porphyrin nanostructures showing both strong surface plasmon resonance and strong luminescence signals. Therefore, the gold–porphyrin assembly continues to show the surface plasmon resonance that is typical of semiconducting Au nanoparticles and that, in contrast, disappears on bulk gold.

As a consequence, the whole Au NPs–porphyrin assembly behaves as a semiconductor, thanks to the extensive electron conjugation granted by the porphyrin molecules that work as the wiring between the different Au NPs.

## 5. Conclusions

To summarize, in the present study, we synthesized nanoscale assemblies of Au nanoparticles self-assembled by means of a new bi-functional porphyrin molecule. In total, 95% of the porphyrin molecules are bound to the surface of gold NPs by one triazine side/leg and some of them, lying close to core-to-core axis, are bridging different gold nanoparticles. This functional architecture exhibits a strong surface plasmon, due to the Au nanoparticles, and a strong luminescence signal coming from porphyrin molecules, thus, giving a new optical material with unique characteristics, similar to those of highly organized networks. In fact, the present network organization continues to grant the Au surface plasmon resonance typical of Au single nanoparticles, while it is well known that bulk Au loses this property, and also grants the porphyrin luminescence. In summary, this artificial Au NPs network may be used for plasmon-enhanced fluorescence, heat generation, photocatalysis, nonlinear optics, solar cells, nanofluidics, photoacoustic, photothermal imaging, cancer therapy, drug delivery, nanotherapeutics, etc., under atmospheric conditions, since our system is not reactive to air nor to water and does not need to be stored in a vacuum or inert gas.

## Supplementary Materials

The following are available online at https://www.mdpi.com/2079-4991/10/9/1644/s1, Figure S1: ^1^H NMR spectrum of the 5,15,-Di(phenyl) 10, 20-Di-benzamide, *N*-ethyl, *N*-1,3,5 Tri-aminotriazine, 21H,23H-porphine recorded in methanol; Figure S2: ^1^H NMR spectrum of the Di-Triazine-Porphyrin, recorded in DMSO-*d_6_*; Figure S3: Selected region of the ^1^H NMR spectrum of the Di-Triazine-Porphyrin after the addition of 10 μL of D_2_O; Figure S4: gCOSY spectrum of the Di-Triazine-Porphyrin recorded in DMSO-*d_6_*; Figure S5: ESI-MS spectrum of Di-Triazine-Porphyrin; Figure S6: EDX spectrum of the Au NPs conjugated with the Di-Triazine-Porphyrin; Figure S7: UV–vis absorbance spectrum of the as synthesized aqueous Au NPs 1.97 × 10^−7^ M solution; Figure S8: UV–vis absorbance spectra of the porphyrin CH_3_OH solution at different concentrations; Figure S9: Trend of the luminescence spectra of the porphyrin CH_3_OH solution at different concentrations; Figure S10: comparison between the PL intensities of the porphyrin CH_3_OH solutions and those obtained during the Au NPs titration at different concentrations; Figure S11: DLS measurements for the 6.57 × 10^−9^ M Au NPs (black line) and 6.22 × 10^−6^ M Au NPs–porphyrin nanostructure methanol solutions (red line). List of the atomic coordinates of the optimized structures of the CL and BL conformers.
