# Supplementary material for: Covalently Conjugated Gold–Porphyrin Nanostructures
Source: Nanomaterials (Basel). 2020 Aug 21;10(9):1644. doi: 10.3390/nano10091644 (PMC7558707; doi:10.3390/nano10091644)
Supplement: Supplementary file 1 [file nanomaterials-10-01644-s001.pdf]

Supplementary Materials

Covalently Conjugated Gold–Porphyrin Nanostructures

Luca Spitaleri [1,2], Chiara M. A. Gangemi [1], Roberto Purrello [1,2], Giuseppe Nicotra [3], Giuseppe Trusso Sfrazzetto [1,2,*] Girolamo Casella [4], Maurizio Casarin [5] and Antonino Gulino [1,2,*]

[1] Department of Chemical Sciences, University of Catania, Viale Andrea Doria 6, 95125 Catania, Italy; luca.spitaleri@phd.unict.it (L.S.); gangemichiara@unict.it (C.M.A.G.); rpurrello@unict.it (R.P.)

[2] National Interuniversity Consortium of Materials Science and Technology (I.N.S.T.M., Research Unit (UdR) of Catania, Viale Andrea Doria 6, 95125 Catania, Italy

[3] National Research Council - Institute for Microelectronics and Microsystems (CNR-IMM), Strada VIII, 5, 95121 Catania, Italy; giuseppe.nicotra@imm.cnr.it (G.N.)

[4] Department of Earth and Sea Sciences, University of Palermo, Via Archirafi 22, 90123 Palermo, Italy; girolamo.casella@unipa.it (G.C.)

[5] Department of Chemical Sciences, University of Padova, Via Francesco Marzolo 1, 35131 Padova, Italy; maurizio.casarin@unipd.it (M.C.)

* Correspondence: giuseppe.trusso@unict.it (G.T.S.); agulino@unict.it (A.G.); Tel.: +39-095-7385067(A.G.); Fax: +39-095-580138 (A.G.)

Received: 06 June 2020; Accepted: 18 August 2020; Published: date

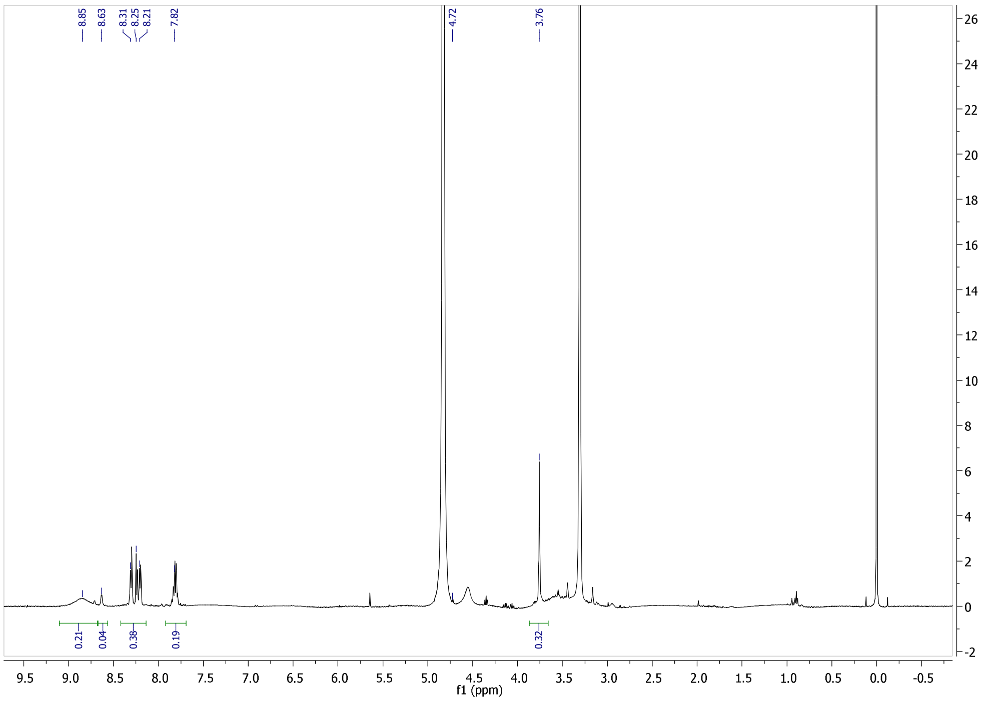

Figure S1. ^1H NMR spectrum of the 5,15,-Di(phenyl) 10, 20 -Di-benzamide, N-ethyl, N- 1,3,5 Tri-aminotriazine, 21H,23H-porphine recorded in methanol.

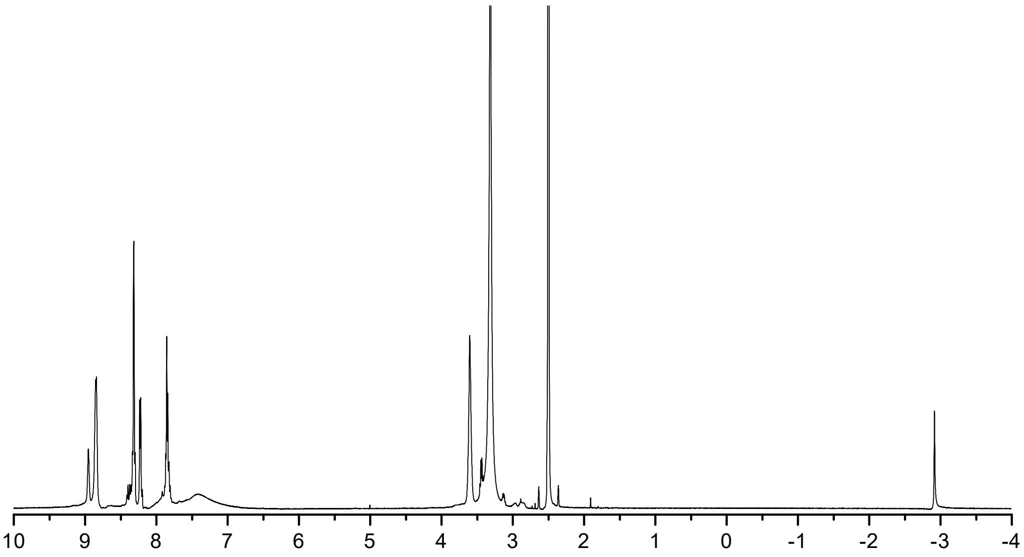

Figure S2. ^1H NMR spectrum of the Di-Triazine-Porphyrin, recorded in DMSO-d_6.

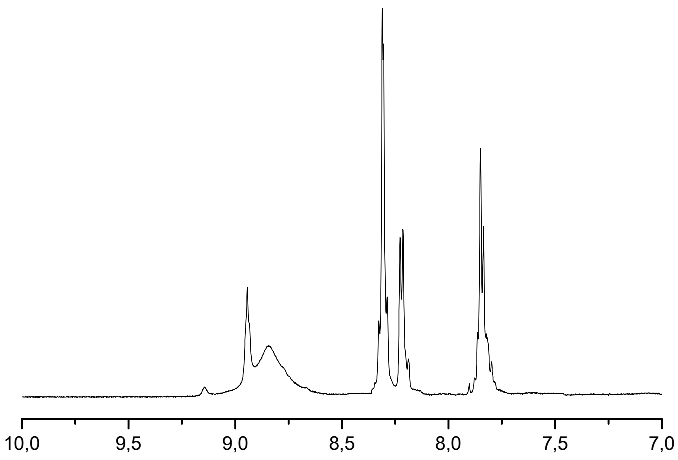

Figure S3. Selected region of the ^1H NMR spectrum of the Di-Triazine-Porphyrin after the addition of 10 μL of D_2O. All broad signals relative to NH-triazine are shifted to 8.84 ppm.

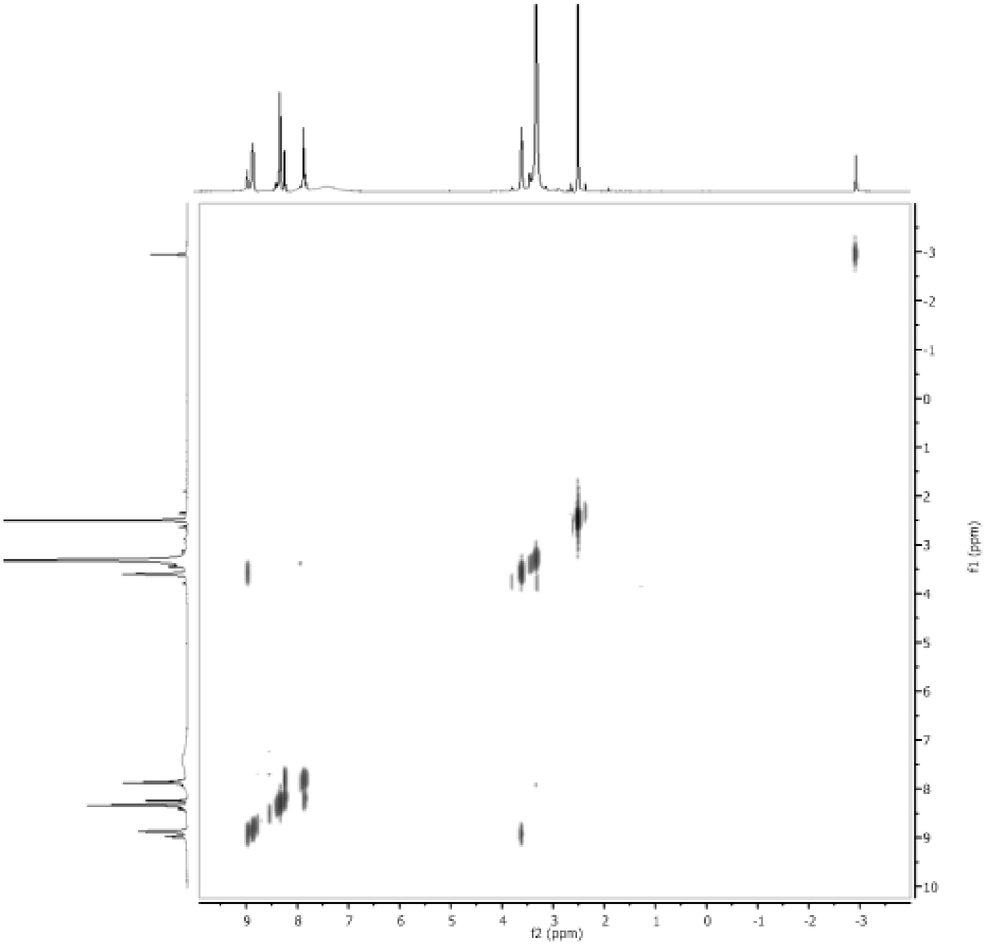

Figure S4. gCOSY spectrum of the Di-Triazine-Porphyrin recorded in DMSO-d_6

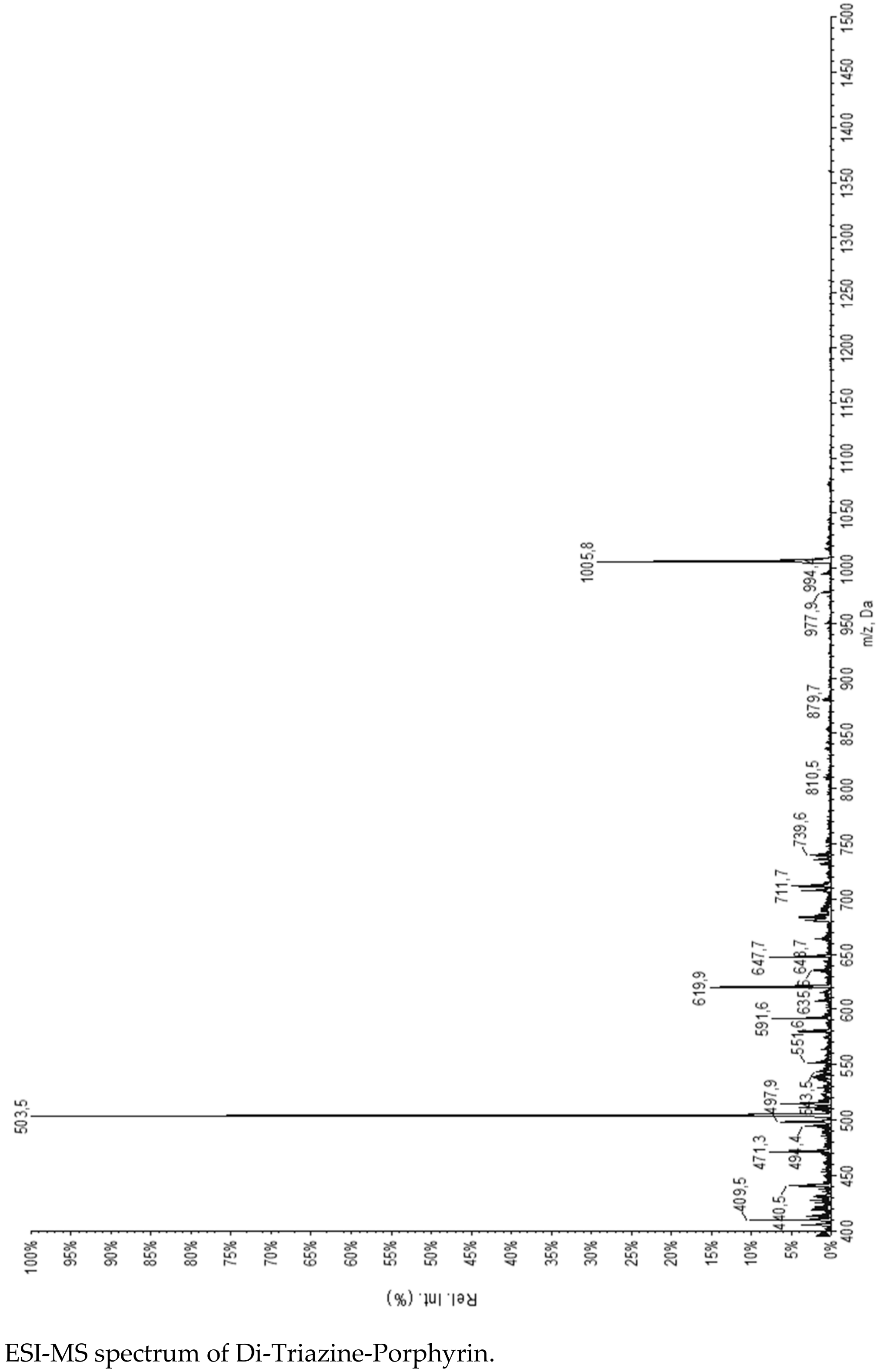

Figure S5. ESI-MS spectrum of Di-Triazine-Porphyrin.

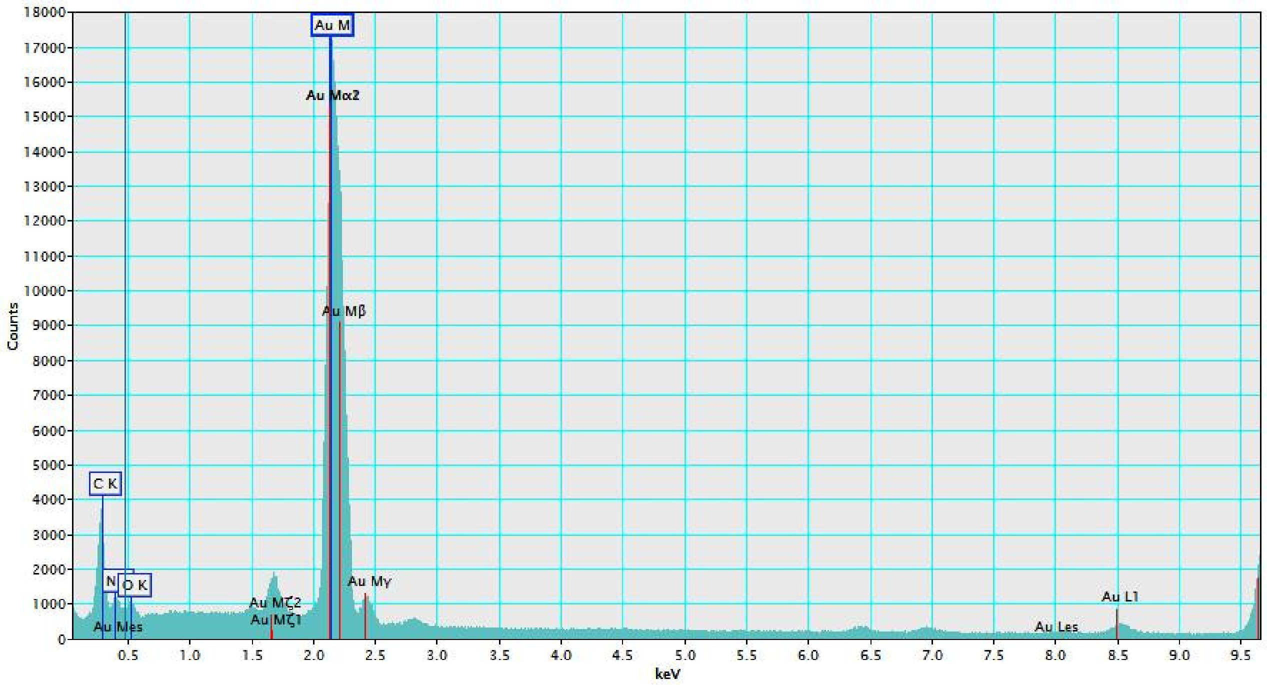

Figure S6. EDX spectrum of the Au NPs conjugated with the 5,15,-Di(phenyl) 10, 20 -Di-benzamide, N-ethyl, N- 1,3,5 Tri-aminotriazine, 21H,23H-porphine.

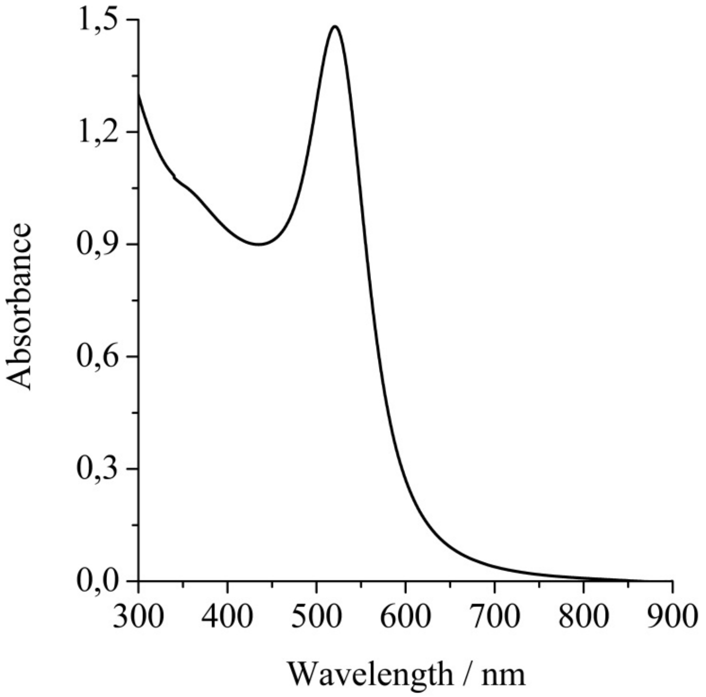

Figure S7. UV-vis absorbance spectrum of the as synthesized aqueous Au NPs $1.97 \cdot 10^{-7}$ M solution.

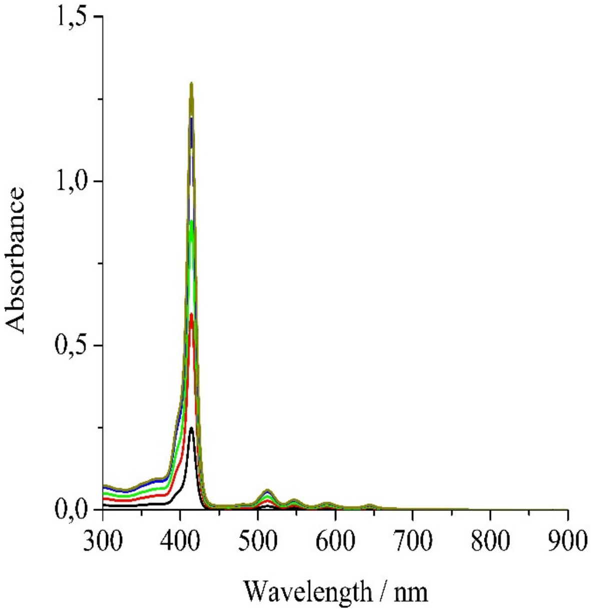

Figure S8. UV-vis absorbance spectra of the porphyrin CH_3OH solution at different concentrations: 9.41·10^{-7} M, black line; 2.13·10^{-6} M, red line; 3.66·10^{-6} M, green line; 5.18·10^{-6} M, blue line; 6.22·10^{-6} M, dark yellow line.

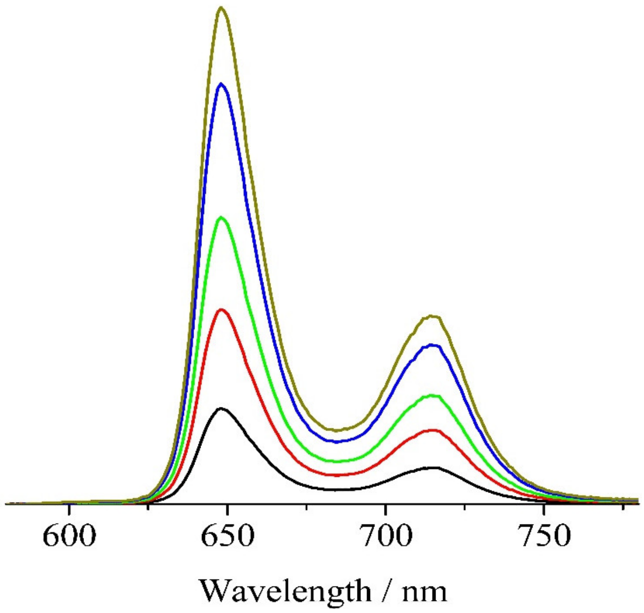

Figure S9. Trend of the luminescence spectra of the porphyrin CH_3OH solution at different concentrations: 9.41·10^{-7} M, black line; 2.13·10^{-6} M, red line; 3.66·10^{-6} M, green line; 5.18·10^{-6} M, blue line; 6.22·10^{-6} M, dark yellow line.

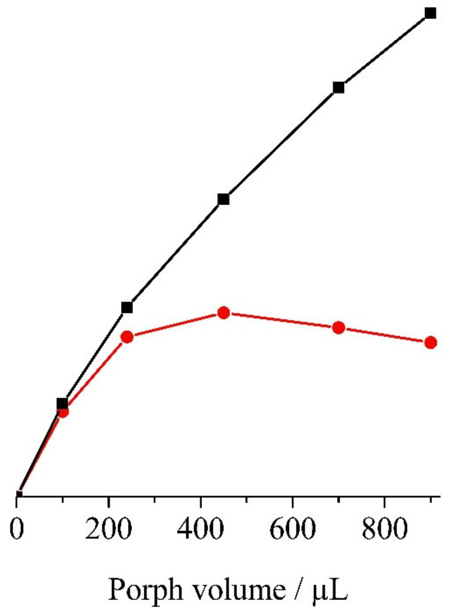

Figure S10. Comparison between the PL intensities of the porphyrin CH_3OH solutions (black line) and those obtained during the Au NPs titration at different concentrations (red line): 9.41·10^{-7} M, black line; 2.13·10^{-6} M, red line; 3.66·10^{-6} M, green line; 5.18·10^{-6} M, blue line; 6.22·10^{-6} M, dark yellow line.

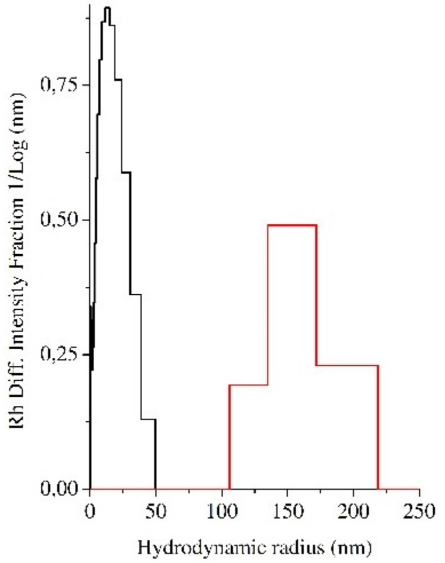

Figure S11. DLS measurements for the 6.57·10^{-9} M Au NPs (black line) and 6.22·10^{-6} M Au NPs – porphyrin nanostructure methanol solutions (red line).

Coordinates of the optimized structures of the LC and LB conformers

CL

122

N	-13.028669	-0.149759	-1.422481
C	-13.543561	-1.064704	-2.284820
N	-14.835185	-0.892469	-2.625438
C	-15.298146	-1.826623	-3.468598
N	-14.605472	-2.864003	-3.974089
C	-13.335067	-2.915736	-3.546507
N	-12.731816	-2.047644	-2.714468
N	-12.584208	-3.961862	-3.986798
N	-16.604460	-1.731019	-3.835376
H	-13.658248	0.592435	-1.159179
H	-12.949979	-4.512179	-4.746218
H	-11.589918	-3.923594	-3.836337
H	-17.088475	-0.873439	-3.626541
H	-16.918917	-2.304676	-4.600407
C	-11.661472	-0.150976	-0.941264
H	-11.362758	-1.179091	-0.720017
H	-11.621709	0.429097	-0.016200
C	7.170762	-1.089890	-0.420436
C	6.405789	-0.037230	-0.943341
C	5.138901	0.235931	-0.438373
C	4.586991	-0.549727	0.590702
C	5.373080	-1.587203	1.128792
C	6.646312	-1.846090	0.635135
C	3.229451	-0.253106	1.100602
C	2.886392	1.162825	1.289229

C	2.332034	-1.257132	1.386918
N	1.134796	-1.016423	2.075359
C	2.436687	-2.678739	1.045101
C	1.311788	-3.261860	1.526249
C	0.534745	-2.184132	2.158551
N	1.727286	1.672394	0.930999
C	3.800948	2.175349	1.837460
C	3.126115	3.350640	1.793114
C	1.812782	3.040842	1.222788
C	0.771009	3.910708	0.987920
C	-0.572015	3.422826	0.647630
N	-1.138667	2.402897	1.255060
C	-1.453197	4.039752	-0.356352
C	-2.606927	3.329070	-0.328171
C	-2.415668	2.289662	0.687697
C	-3.306041	1.312792	1.072207
C	-2.896861	0.191832	1.929125
N	-1.770995	-0.467264	1.757187
C	-3.003901	-1.403175	3.523559
C	-3.707614	-0.355950	3.026632
C	-0.742049	-2.379151	2.857991
C	-1.776859	-1.479417	2.726945
C	-4.722898	1.343702	0.644695
C	-5.478011	2.532401	0.657082
C	-6.808687	2.535927	0.255166
C	-7.423984	1.358250	-0.187066
C	-6.687180	0.165002	-0.176853
C	-5.361506	0.155463	0.243154
C	8.555418	-1.434684	-0.900825
O	9.352313	-2.024600	-0.177353

N	8.850883	-1.058327	-2.185327
C	10.142105	-1.316745	-2.802721
C	0.953050	5.377812	1.057816
C	-0.028938	6.181664	1.668347
C	0.131243	7.562200	1.750769
C	1.264028	8.171882	1.206185
C	2.235713	7.390922	0.578169
C	2.083315	6.007764	0.503624
C	-0.866544	-3.590485	3.699122
C	0.208515	-3.997002	4.512909
C	-2.034865	-4.375714	3.692618
C	0.105227	-5.129734	5.315682
C	-1.064926	-5.892765	5.306826
C	-2.131354	-5.514995	4.489212
H	6.808695	0.601235	-1.724350
H	4.558384	1.055357	-0.849051
H	4.985817	-2.168613	1.958263
H	7.264643	-2.628015	1.062303
H	3.223971	-3.138358	0.465081
H	1.011623	-4.298341	1.446716
H	4.794578	1.991328	2.224581
H	3.446436	4.312519	2.166695
H	-1.198912	4.873501	-0.997425
H	-3.471880	3.446239	-0.965109
H	-3.246414	-2.018134	4.378177
H	-4.655065	0.035307	3.373374
H	-5.018056	3.447611	1.013480
H	-7.401045	3.443949	0.285445
C	-8.864371	1.443792	-0.611730
H	-7.153211	-0.773988	-0.461748

H	-4.803332	-0.774860	0.249890
H	8.098931	-0.723332	-2.767781
H	10.726445	-1.902172	-2.092125
H	10.009839	-1.907816	-3.717579
H	-0.906666	5.710151	2.098336
H	-0.628787	8.163747	2.240437
H	1.384036	9.249702	1.263701
H	3.110049	7.860008	0.136584
H	2.826146	5.406115	-0.008898
H	1.116169	-3.402352	4.526806
H	-2.853749	-4.099437	3.037294
H	0.938692	-5.418179	5.949255
H	-1.141423	-6.780910	5.927126
H	-3.037089	-6.113740	4.463695
O	-9.596797	2.353951	-0.227966
N	-9.308773	0.463517	-1.454154
C	-10.676633	0.460245	-1.955693
H	-8.645413	-0.184315	-1.847770
H	-10.707842	-0.120919	-2.879774
H	-10.971804	1.490793	-2.174254
C	10.896935	-0.012498	-3.124810
N	12.167499	-0.243527	-3.783681
C	13.296165	-0.648092	-3.138365
N	13.217950	-0.823945	-1.811319
N	14.385957	-0.832117	-3.907162
C	15.465829	-1.226556	-3.214032
N	15.531192	-1.452271	-1.889672
C	14.372363	-1.226205	-1.250476
N	14.361864	-1.404155	0.096861
N	16.618325	-1.397960	-3.918186

H	16.549495	-1.434799	-4.921946
H	17.374906	-1.877858	-3.458791
H	11.068512	0.531118	-2.191961
H	10.291928	0.624626	-3.779848
H	12.250580	-0.161118	-4.784650
H	15.144281	-1.885567	0.508984
H	13.463350	-1.443942	0.551331

BL

122

N	-12.727846	0.380565	2.813752
C	-13.814085	-0.208186	2.247587
N	-14.942820	-0.193724	2.982108
C	-15.988534	-0.770500	2.372499
N	-15.996882	-1.318652	1.143064
C	-14.805775	-1.268491	0.528841
N	-13.672366	-0.734439	1.017635
N	-14.728585	-1.840337	-0.705641
N	-17.154563	-0.825338	3.070778
H	-12.861047	0.701621	3.760438
H	-15.598153	-2.058757	-1.164631
H	-13.925032	-1.615303	-1.269230
H	-17.230007	-0.260334	3.900601
H	-17.988658	-1.079058	2.567462
C	-11.408354	0.414825	2.214818
H	-10.839092	1.224196	2.677681
H	-11.513188	0.628647	1.147607
C	7.698568	0.499781	-0.879359

C	6.876449	-0.601080	-0.597619
C	5.502039	-0.526888	-0.798151
C	4.903395	0.657691	-1.266784
C	5.739452	1.749530	-1.570934
C	7.114363	1.667488	-1.385623
C	3.437548	0.722118	-1.463630
C	2.797770	-0.448348	-2.077510
C	2.712234	1.832709	-1.093912
N	1.377665	2.019505	-1.479622
C	3.160052	2.961320	-0.272950
C	2.097643	3.797200	-0.176548
C	1.013989	3.166132	-0.946410
N	1.652902	-0.942086	-1.655746
C	3.375453	-1.230772	-3.180382
C	2.507307	-2.246284	-3.412166
C	1.414069	-2.069535	-2.453065
C	0.289264	-2.850903	-2.305838
C	-0.843978	-2.416294	-1.478855
N	-1.309214	-1.185396	-1.490319
C	-1.622219	-3.303183	-0.600229
C	-2.605678	-2.535234	-0.070517
C	-2.413375	-1.193093	-0.627070
C	-3.163286	-0.064538	-0.379937
C	-2.712967	1.268213	-0.799170
N	-1.466722	1.674587	-0.676410
C	-2.804448	3.395715	-1.549867
C	-3.593176	2.312185	-1.344102
C	-0.310939	3.763019	-1.157732
C	-1.457694	2.999739	-1.131270
C	-4.469326	-0.140642	0.313250

C	-5.409287	-1.140695	0.003933
C	-6.639918	-1.188444	0.652974
C	-6.968138	-0.238545	1.629714
C	-6.031299	0.753062	1.951431
C	-4.810198	0.813159	1.292678
C	9.192549	0.493020	-0.706559
O	9.913047	1.283562	-1.313490
N	9.702982	-0.440224	0.154326
C	11.132108	-0.508582	0.427039
C	0.168279	-4.169997	-2.966201
C	-1.049228	-4.550963	-3.562669
C	-1.177711	-5.785377	-4.193578
C	-0.100764	-6.674628	-4.223463
C	1.106081	-6.320233	-3.618235
C	1.241061	-5.080882	-2.995450
C	-0.356715	5.223801	-1.391284
C	0.595092	5.833529	-2.231333
C	-1.321191	6.039638	-0.770252
C	0.562125	7.205277	-2.466714
C	-0.407791	8.000495	-1.851591
C	-1.344939	7.414057	-0.999323
H	7.307159	-1.537018	-0.253848
H	4.877100	-1.386407	-0.579386
H	5.301762	2.652823	-1.981525
H	7.764860	2.497072	-1.640267
H	4.122616	3.055622	0.208742
H	2.028131	4.726869	0.372546
H	4.292371	-1.003846	-3.708269
H	2.557130	-2.995882	-4.188723
H	-1.414016	-4.347219	-0.406899

H	-3.340368	-2.818307	0.669596
H	-3.079367	4.339642	-1.997987
H	-4.647924	2.205345	-1.560690
H	-5.174397	-1.869552	-0.763878
H	-7.331336	-1.989689	0.408733
C	-8.272823	-0.229839	2.378065
H	-6.285632	1.466388	2.727671
H	-4.095178	1.588727	1.546791
H	9.073990	-0.914976	0.783583
H	11.660331	-0.397062	-0.523003
H	11.361331	-1.492447	0.843205
H	-1.885429	-3.859453	-3.546050
H	-2.119454	-6.054731	-4.662538
H	-0.204644	-7.640850	-4.708288
H	1.941807	-7.013797	-3.622091
H	2.170859	-4.819205	-2.502116
H	1.345849	5.217320	-2.715411
H	-2.032234	5.590336	-0.085465
H	1.294278	7.655103	-3.130720
H	-0.427258	9.071749	-2.028973
H	-2.090213	8.028806	-0.503231
O	-8.385360	0.328820	3.467623
N	-9.319464	-0.881818	1.784496
C	-10.638716	-0.907673	2.401108
H	-9.244626	-1.130949	0.810282
H	-10.507915	-1.106812	3.467610
H	-11.213296	-1.724958	1.958796
C	11.602229	0.583649	1.407816
N	13.026555	0.523276	1.668752
C	13.615170	-0.340906	2.536871

N	12.816538	-1.152572	3.253010
N	14.959352	-0.286732	2.601839
C	15.490026	-1.145036	3.484599
N	14.815491	-1.998466	4.277387
C	13.486442	-1.950085	4.103835
N	12.739648	-2.815033	4.845910
N	16.847067	-1.171656	3.577578
H	17.359367	-0.418884	3.148061
H	17.251278	-1.661897	4.358457
H	11.067613	0.479057	2.355858
H	11.370269	1.563148	0.983284
H	13.665293	1.065534	1.107476
H	13.196901	-3.267648	5.620840
H	11.749276	-2.641185	4.899885
